# Supplementary material for: Tumor-Promoting Actions of HNRNP A1 in HCC Are Associated with Cell Cycle, Mitochondrial Dynamics, and Necroptosis
Source: Int J Mol Sci. 2022 Sep 6;23(18):10209. doi: 10.3390/ijms231810209 (PMC9499416; doi:10.3390/ijms231810209)
Supplement: Supplementary file 1 [file ijms-23-10209-s001.zip › ijms-1878576-supplementary.pdf]

SUPPLEMENTARY MATERIALS

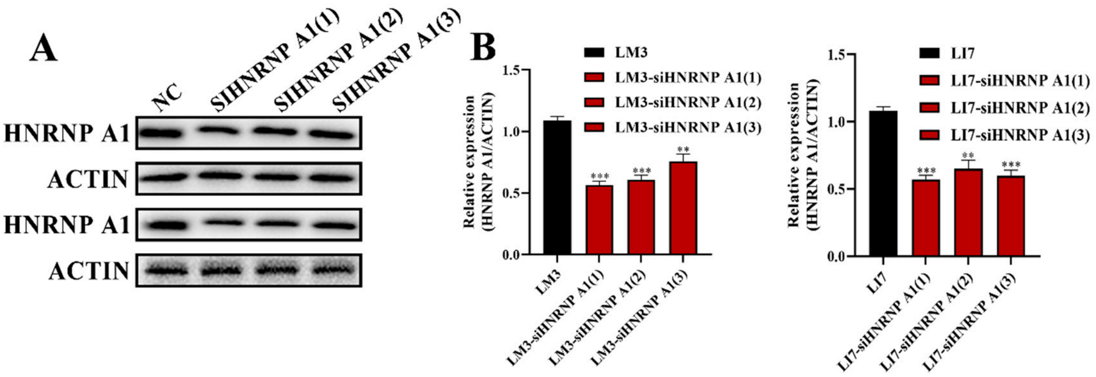

Supplementary Figure S1. Knockout efficiency of HNRNP A1.

A. The expression of HNRNP A1 After lentivirus were respectively constructed to infect LM3 cells (up) and LI7 cells (down) detected by WB. B. The expression of HNRNP A1 After lentivirus were respectively constructed to infect LM3 cells (up) and LI7 cells (down) detected by qPCR. *p< 0.05; **p< 0.01; ***p< 0.001. The data expressed as the mean ± SD.

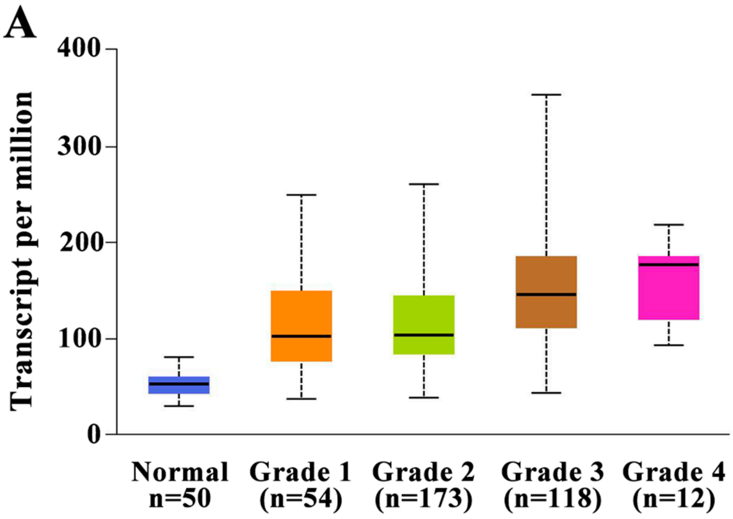

Supplementary Figure S2. Expression of HNRNP A1 in different HCC grade in TCGA.

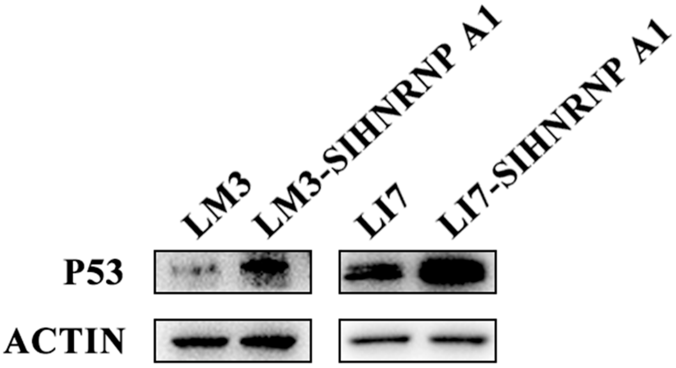

Supplementary Figure S3. Expression of P53 after knocking down HNRNP A1.
